# Solamargin-functionalized gold nanoparticles codelivering photothermal-immunotherapy for triple-negative breast cancer

**DOI:** 10.1007/s12282-026-01849-0

**Published:** 2026-04-01

**Authors:** Zhengwei Gui, Lu Zhao, Shiyang Liu, Lin Zhang

**Affiliations:** Department of Thyroid and Breast Surgery, Tongji Hospital of Tongji Medical College of Huazhong, University of Science and Technology, Wuhan, 430030 Hubei Province China

**Keywords:** solamargin, gold nanoparticles, breast cancer, immune microenvironment, photothermal therapy

## Abstract

**Background:**

The high rates of metastasis and recurrence in triple-negative breast cancer (TNBC) underscore the limitations of current therapies. Solamargin (SM), a bioactive glycoside, possesses potential antitumor activity, but its efficacy is limited by low potency and off-target effects.

**Methods:**

We engineered Au@PEG-SM, a nanoconjugate designed for targeted delivery. Beyond traditional direct killing, we employed vascular endothelial cell models, STING-specific inhibitors (H-151), and cytokine neutralization assays to rigorously validate the molecular mechanism. Systemic immune profiling, including the analysis of lymph node dendritic cells, and bilateral tumor models were used to assess antitumor efficacy.

**Results:**

Au@PEG-SM synergizes with photothermal therapy (PTT) to achieve a potent 1 plus 1 greater than 2 therapeutic effect. Mechanistically, low-dose SM sensitizes the cGAS-STING pathway in vascular endothelial cells; upon synergistic activation by PTT-released DNA, this triggers a massive secretion of IFN-β and TNF-α, leading to indirect tumor cell apoptosis. This process, combined with robust immunogenic cell death (ICD) and promoted dendritic cell maturation in lymph nodes, triggers a systemic antitumor response. Comprehensive immune profiling revealed that this combination therapy significantly increases the infiltration of NK cells and CD8 + T cells while markedly reducing immunosuppressive MDSCs and regulatory T cells (Tregs) in both primary and distant tumors. When combined with anti-PD-L1 blockade, the therapy eradicated primary tumors and established durable immune memory.

**Conclusions:**

This study establishes Au@PEG-SM as a powerful platform that achieves systemic immune activation via a sensitized STING-secretome-DC cascade. By providing rigorously validated mechanistic insights and a holistic immune landscape, our work offers a promising multimodal paradigm for overcoming TNBC.

**Graphical abstract:**

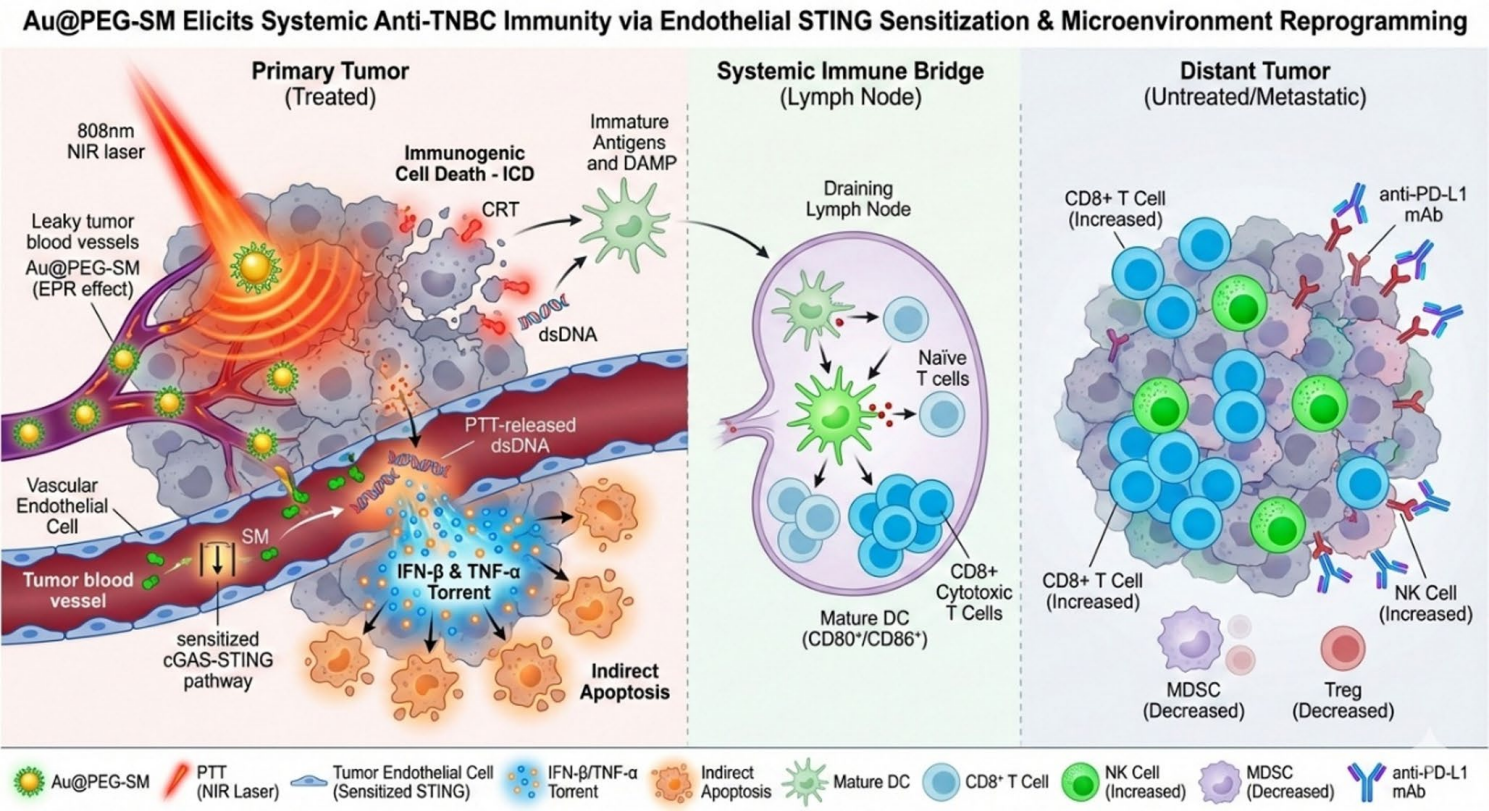

## Background

Breast cancer (BC) represents one of the most common and serious health threats to women globally [1]. With a rising incidence worldwide, particularly in high-income countries where approximately one in eight women is affected, BC poses a significant public health challenge [2]. Standard treatment modalities include surgery, endocrine therapy, chemotherapy, radiotherapy, and targeted therapy. Among BC subtypes, TNBC presents major therapeutic challenges due to its lack of specific molecular targets and high rates of metastasis and recurrence [3]. Defined by the absence of estrogen receptor, progesterone receptor, and HER2 receptor expression, TNBC is resistant to conventional endocrine and HER2-targeted treatments. Therefore, developing novel therapeutic strategies to control TNBC progression and prevent disease recurrence is of critical importance [4].

Solamargin (SM), a natural alkaloid derived from plants of the Solanaceae family, has recently gained increasing attention for its antitumor potential. Studies have demonstrated its remarkable anticancer efficacy across various cancer types. For instance, SM enhances the antitumor effect of sorafenib in hepatocellular carcinoma by regulating the HOTTIP-TUG1/miR-4726-5p/MUC1 signaling axis [5]. Similarly, in non-small cell lung cancer (NSCLC), SM augments the efficacy of EGFR-TKIs (e.g., erlotinib and gefitinib) through modulation of the MALAT1/miR-141-3p/Sp1/IGFBP1 pathway [6]. However, the clinical translation of many traditional Chinese medicine (TCM) monomers, including SM, is often hampered by dose-limiting toxicities [7, 8]. For example, cinnabar, a mineral-based TCM, exerts markedly different effects on the mouse cerebral cortex at therapeutic versus toxic doses, suggesting distinct molecular mechanisms at different concentrations [9]. Therefore, strategies that enable tumor-selective accumulation of active TCM components while minimizing systemic exposure are crucial for enhancing both the safety and efficacy of these agents [10–12].

Gold nanoparticles (AuNPs) can passively accumulate in tumor tissues through the enhanced permeability and retention (EPR) effect. Furthermore, owing to their exceptional photothermal conversion efficiency, AuNPs have emerged as a prominent agent for PTT. While prior studies have explored the role of endothelial STING activation in cancer cell death, the challenge remains to achieve such activation safely without high systemic toxicity. Our work differentiates itself by utilizing a low-dose SM delivery platform to achieve this effect. Building upon the established role of the cGAS-STING pathway in regulating the tumor microenvironment, we developed Au@PEG-SM, a nanoconjugate composed of PEG-modified AuNPs covalently linked with SM, for multimodal synergistic therapy against TNBC. Upon intravenous administration, Au@PEG-SM preferentially accumulated in tumor tissue. We hypothesized that within this specialized delivery platform, the pharmacological action of low-dose SM could further synergize with AuNP-mediated PTT to activate the cGAS signaling pathway and induce robust ICD. This, in turn, enhanced the infiltration of CD8⁺ T cells, increased the M1/M2 macrophage ratio, and reduced the population of regulatory T cells (Tregs) within the primary tumor. The subsequent release of tumor antigens and the maturation of dendritic cells (DCs) initiated a potent systemic antitumor immune response in our experimental model. When combined with anti-PD-L1 checkpoint blockade, this Au@PEG-SM–mediated strategy demonstrated the potential to eradicate both primary and distant tumors, while conferring protection against tumor recurrence.

## Methods

### Details of the experimental model and subjects are provided

The study utilized female BALB/c mice (aged 6–8 weeks, average weight 20 g) supplied by GemPharmatech Co., Ltd (China). All mice were maintained in a specific pathogen-free (SPF) environment at 22 °C ± 1 °C and 50% ± 1% relative humidity, with a standard 12-hour light/dark cycle. All experimental procedures were conducted in strict accordance with the standard operating protocols of the Laboratory Animal Center at Tongji Hospital, Tongji Medical College, Huazhong University of Science and Technology. The study protocol received formal approval from the center’s Animal Welfare and Ethics Committee (Approval Document: TJH-243688).

To ensure scientific rigor and minimize potential confounders, mice were randomly assigned to experimental groups using a random number table once the primary tumor volume reached approximately 100 mm³. For outcome assessments, tumor volume measurements and histopathological evaluations were performed by researchers blinded to the treatment assignments.

The sample sizes were determined based on the requirements of statistical power and the 3Rs principle (Reduction, Replacement, and Refinement): *n* = 10 was utilized for long-term survival analysis to account for potential variability and ensure sufficient statistical power over the observation period. *n* = 6 was employed for monitoring tumor growth kinetics to provide reliable data for trend analysis while minimizing animal usage. *n* = 3 to 5 was selected for mechanistic studies, including fluorescence imaging, flow cytometry, and ELISA, where intra-group variance is typically low.

### Materials

Chlorogenic acid, sodium citrate, EDC/NHS, dimethyl sulfoxide (DMSO), and HS-PEG-COOH were purchased from Sigma-Aldrich Life Sciences and High Technology Group, Inc. Australian solanine (with high-performance liquid chromatography purity ≥ 98%) was obtained from Chengdu Mence Biotechnology Co., Ltd.

### Preparation of Au@PEG-COOH

First, AuNPs were synthesized by rapidly adding 2 mL of a 1% (w/v) trisodium citrate solution to 100 mL of boiling 0.01% (w/v) chloroauric acid under vigorous magnetic stirring. The reaction was maintained under boiling conditions for 10–15 min before being cooled to room temperature with continuous stirring. Next, 10 mL of the resulting AuNP solution was mixed with 0.5 mg of HS-PEG-COOH and vigorously stirred for 12 h at room temperature in the dark. Finally, the Au@PEG-COOH NPs were collected by centrifugation at 10,000 × g for 10 min and washed three times with ultrapure water to remove unreacted reagents.

### Preparation of Au@PEG-SM

To conjugate SM to the nanoparticles, the terminal carboxyl groups of PEG were first activated. One milliliter of Au@PEG-COOH was incubated with 20 µL each of freshly prepared EDC and NHS (100 mM) for 30 min at room temperature in the dark, thereby converting the carboxyls into reactive NHS esters. Separately, an SM stock solution was prepared by dissolving the compound in anhydrous DMSO at a concentration of 1 mg/mL. Then, 50 µL of this SM solution was added to the activated nanoparticle mixture and allowed to react under gentle stirring at 4 °C for 12 h in the dark. The final Au@PEG-SM conjugates were collected and cleaned via triple centrifugation/washing cycles (10,000 × g, 10 min) using ultrapure water.

### Characterization

The morphology of the synthesized Au@PEG-SM was investigated with a JEOL JEM-1200EX transmission electron microscope (Tokyo, Japan). Meanwhile, the hydrodynamic diameter and surface charge (zeta potential) of the Au@PEG and Au@PEG-SM nanoparticles were determined by dynamic light scattering (DLS) on a Zetasizer Nano ZS90 system (Malvern, UK).

### Pharmacokinetics and Biodistribution Analysis

For the pharmacokinetic study, 4T1 tumor-bearing mice were intravenously injected with Au@PEG-SM (*n* = 3). Blood samples (approximately 20 µL) were collected via the tail vein at designated time points (0.5, 1, 4, 8, 12, 24, and 48 h) post-injection. Each blood sample was immediately mixed with heparin to prevent coagulation and then subjected to acid digestion.

For the biodistribution analysis, mice were sacrificed at 24 h post-injection. Major organs (heart, liver, spleen, lung, and kidney) and tumor tissues were harvested, rinsed with PBS, and weighed. All collected biological samples (blood and tissues) were digested in a mixture of concentrated HNO3 and HClO4 (v/v = 3:1) at 120 degrees Celsius for 4 h until a clear, colorless solution was obtained. After cooling to room temperature, the digested solutions were diluted to a final volume of 10 mL with 2% HNO3 and filtered through a 0.22 μm membrane.

The gold (Au) concentration in each sample was quantified using Inductively Coupled Plasma Mass Spectrometry (ICP-MS, Agilent 7800, USA). A series of Au standard solutions (0, 0.5, 1, 5, 10, 50, and 100 ng/mL) were used to establish the calibration curve. The results were expressed as micrograms of Au per milliliter of blood (µg/mL) or micrograms of Au per gram of tissue (µg/g tissue).

Validation of Synergistic Endothelial STING Pathway Activation.

To investigate the synergistic activation and the underlying mechanism of the STING pathway, vascular endothelial cells were assigned to six experimental groups: (1) Control (untreated), (2) PTT Ligand (DNA only), (3) SM Only, (4) Synergy (SM + DNA), (5) Synergy + DNase I (G4 treated with DNase I), and (6) Pathway Blockade (G4 treated with H-151). For the blocking groups, cells were pre-incubated with DNase I (to degrade DNA ligands) or H-151 (10 µM, a STING inhibitor) for 2 h prior to the synergistic treatment.

For Western blot analysis, total proteins were extracted from cells in different groups using RIPA lysis buffer containing protease and phosphatase inhibitors. Protein concentrations were quantified using the BCA assay. Equal amounts of protein (30 µg) were separated by SDS-PAGE and transferred onto PVDF membranes. The membranes were blocked with 5% non-fat milk and incubated overnight at 4 degrees Celsius with primary antibodies against p-STING (Ser366), STING, p-IRF3 (Ser396) and GAPDH. Following incubation with HRP-conjugated secondary antibodies, the protein bands were detected using an enhanced chemiluminescence (ECL) kit and analyzed with ImageJ software.

For RT-qPCR analysis, total RNA was isolated using Trizol reagent and reverse-transcribed into cDNA. The expression levels of IFN-β and CXCL10 mRNA were determined using a SYBR Green PCR kit, with GAPDH serving as the internal reference. Furthermore, the concentrations of secreted IFN-β and TNF-α in the culture supernatants were quantified using commercial ELISA kits according to the manufacturer’s instructions.

### Cytokine Neutralization and Indirect Tumor Cell Killing Assay

To confirm the role of specific cytokines in the indirect tumoricidal effect, a cytokine neutralization assay was performed. Conditioned media (CM) were collected from vascular endothelial cells after the indicated synergistic treatments (SM + DNA) and centrifuged at 1000 g for 5 min to remove cell debris. 4T1 tumor cells were then cultured in the collected CM for 24 h.

To determine which cytokines were responsible for tumor cell death, neutralizing antibodies against IFN-β or TNF-α (10 µg/mL) were added to the CM either individually or in combination before being applied to the tumor cells. A non-specific IgG antibody was used as an isotype control.

The viability of 4T1 cells was evaluated using the MTT assay. Briefly, 20 µL of MTT solution (5 mg/mL) was added to each well and incubated at 37 degrees Celsius for 4 h. After the supernatant was carefully removed, 150 µL of DMSO was added to each well to dissolve the purple formazan crystals. The plate was then shaken for 10 min, and the absorbance was measured at 570 nm using a microplate reader.

Furthermore, tumor cell apoptosis was assessed using an Annexin V-FITC/PI apoptosis detection kit. After 24 h of incubation with different CM, the 4T1 cells were harvested, stained according to the manufacturer’s instructions, and analyzed by flow cytometry (BD FACSCalibur, USA). The percentage of apoptotic cells was calculated using FlowJo software.

### In vitro study

#### Cell culture

The human breast cancer cell line MDA-MB-231, the mouse breast cancer cell line 4T1, and the human umbilical vein endothelial cell line (HUVEC) were purchased from NuwaCell Biotechnology Co., Ltd. (Hefei, China). Cells were propagated under standard conditions (37 °C, 5% CO₂). MDA-MB-231 cells were cultured in Dulbecco’s Modified Eagle Medium (DMEM); 4T1 cells were cultured in Roswell Park Memorial Institute (RPMI)-1640 medium; and HUVECs were cultured in their designated endothelial cell medium. All media were sourced from Gibco and supplemented with 10% fetal bovine serum (FBS). Cells were kept at subconfluent density (50–80%), and only those in the logarithmic phase of growth were used for subsequent assays.

mRNA-sequencing.

Total RNA was isolated from treated and untreated HUVECs using the RNeasy kit (QIAGEN). RNA integrity was verified using an Agilent 2100 Bioanalyzer, and all samples had an RNA Integrity Number (RIN) > 8.0. Sequencing libraries were prepared from 1 µg of total RNA using the Illumina TruSeq Stranded mRNA LT Sample Prep Kit and sequenced on an Illumina NovaSeq 6000 platform to generate 150 bp paired-end reads. The raw sequencing data are available upon reasonable request from the corresponding author. After quality control with FastQC (v0.12.1), differential expression analysis was performed using the DESeq2 package (v1.40.0) with a threshold of |log2FoldChange| > 1.5 and an adjusted p-value < 0.05. Functional enrichment analysis of differentially expressed genes (DEGs) for KEGG pathways and Gene Ontology (GO) terms was conducted using the ClusterProfiler package (v4.10.0).

### ELISA

The concentrations of IFN-β, TNF-α, and cGAMP were determined using commercially available ELISA kits following the manufacturers’ instructions. For IFN-β (PBL Assay Science, Cat# 41410) and TNF-α (BioLegend, Cat# 430904), a standard sandwich ELISA procedure was employed: plates were incubated with samples/standards, followed by detection antibodies and streptavidin-HRP. For cGAMP (Arigo Biolaboratories, Cat# ARG82951), a competitive ELISA format was used, where samples competed with a plate-bound conjugate for binding to a specific antibody. In all cases, after the addition of TMB substrate and stopping the reaction, the absorbance was measured at 450 nm. The exact incubation times and temperatures were as prescribed by each kit.

### In vivo study

In vivo biocompatibility Assessment.

To evaluate the biocompatibility of the treatment, major organs (including the heart, liver, spleen, lungs, and kidneys) were harvested, fixed in 10% neutral buffered formalin, and processed for paraffin embedding. The embedded tissues were sectioned into 5 μm slices and stained with hematoxylin and eosin (H&E) for histological examination under a light microscope. Furthermore, blood samples were collected, and serum was separated for the analysis of biochemical parameters using an automated biochemical analyzer (Chemray 420).

### In vivo imaging

To quantitatively evaluate the biodistribution profile, mice (*n* = 5 per group) were intravenously injected with Au@PEG-SM-DiD (10 mg SM/kg) via the tail vein. Whole-body fluorescence imaging was performed at predetermined time points (0, 3, 6, 12,24, 36,48and 72 h post-injection) using the IVIS^®^ Lumina Series III imaging system (PerkinElmer, USA). During imaging, mice were anesthetized with isoflurane and positioned in a standardized, supine orientation. Fluorescence signals were acquired with consistent parameters (exposure time: 1 s, f/stop: 2, binning: medium). The resulting radiant efficiency was quantified within defined regions of interest (ROIs) over major organs using Living Image^®^ software (v4.5). Data are presented as the mean ± standard deviation (SD), and statistical significance between groups at specific time points was determined by one-way ANOVA followed by Tukey’s post-hoc test. A p-value < 0.05 was considered statistically significant.

### Quantitative Biodistribution and Pharmacokinetics

For quantitative analysis, gold (Au) content was measured using Inductively Coupled Plasma Mass Spectrometry (ICP-MS, Agilent 7700). For the biodistribution study, mice (*n* = 3) were sacrificed 24 h post-injection; major organs (heart, liver, spleen, lung, and kidney) and tumors were harvested, weighed, and digested in aqua regia. For the pharmacokinetic study, blood samples (50 µL) were collected from the tail vein at predetermined intervals (0.5, 1, 2, 4, 8, 12, 24, and 48 h). After digestion, the Au concentration in each sample was determined. Pharmacokinetic parameters, including the blood circulation half-life (t1/2), were calculated using a two-compartment model via Phoenix WinNonlin software.

### Immunofluorescence staining

For immunofluorescence staining, frozen tumor sections or fixed cells were first fixed in 4% paraformaldehyde (20 min, RT) and permeabilized with 1% Triton X-100 (Solarbio; 15 min, RT). Afterward, samples were blocked for 1 h at RT with a solution of 5% BSA and 0.1% Triton X-100 in PBS to minimize nonspecific background. The samples were then incubated overnight at 4 °C with specific primary antibodies diluted in blocking buffer. The primary antibodies used were: rabbit anti-Calreticulin (CRT, Abcam ab191014, 1:200), rabbit anti-phospho-STING (p-STING, Abcam ab239074, 1:150), and rabbit anti-PD-L1 (Abcam ab279294, 1:250). The following day, samples were washed thoroughly with PBS and incubated for 1 h at RT in the dark with Alexa Fluor-conjugated secondary antibodies ( Goat anti-Rabbit 488, 1:500). After final washes, cell nuclei were stained with DAPI contained within ProLong Diamond Antifade Mountant (Invitrogen, P36962), and coverslips were applied. Fluorescence images were captured using a Leica Stellaris 5 confocal laser scanning microscope with consistent settings across compared groups.

### qRT-PCR

Total RNA was extracted using the GeneJET RNA Purification Kit. cDNA was synthesized from 1 µg of total RNA with the Invitrogen Superscript cDNA Synthesis Kit (Thermo Fisher Scientific) according to the manufacturer’s instructions. Quantitative real-time PCR (qRT-PCR) was performed using SYBR Green master mix on a QuantStudio 5 Real-Time PCR System (Applied Biosystems). The following primer sequences were used for amplification: IFN-β1 (forward: 5′-CTGCTGGTTGCAGCTCTAAAC-3′, reverse: 5′-GACATTAGCCAGGAGGTTCTCA-3′), TNF-α (forward: 5′-CCTCTCTCTAATCAGCCCTCTG-3′, reverse: 5′-GAGGACCTGGGAGTAGATGAG-3′), CXCL-9 (forward: 5′-GACGGCCCTGGTGCTAGT-3′, reverse: 5′-CCTTTTCGGATCACTTCGCTT-3′), ICAM-1 (forward: 5′-CTGTGTGTCCAGCTCCAAGA-3′, reverse: 5′-TGGGTCTCTGCTGGTGAATC-3′), VCAM-1 (forward: 5′-CCTCACTTGACCTCTTCCTG-3′, reverse: 5′-CACTTGAAGTGTCTCCTGGC-3′), CXCL-10 (forward: 5′-GTGGCATTCAAGGAGTACCTC-3′, reverse: 5′-TGATGGCCTTCGATTCTGGATT-3′), and GAPDH (forward: 5′-GGAGCGAGATCCCTCCAAAAT-3′, reverse: 5′-GGCTGTTGTCATACTTCTCATGG-3′). Gene expression was normalized to GAPDH and calculated using the comparative 2^–ΔΔCT method.

### Immune cell ratio analysis

To analyze immune cell populations, single-cell suspensions were prepared from murine inguinal lymph nodes, tumors, and spleens. The tissues were mechanically dissociated and enzymatically digested at 37 °C for 30 min in RPMI-1640 medium containing collagenase type IV (2 mg/mL) and DNase I (50 µg/mL) with gentle agitation. The resulting cell suspensions were passed through a 70 μm cell strainer and washed with PBS. After red blood cell lysis (for spleen samples), the cells were counted and viability was assessed. To ensure data reliability, doublets were excluded using FSC-H/FSC-A and SSC-H/SSC-A parameters, and dead cells were excluded by staining with a fixable viability dye (Zombie Aqua, BioLegend). Fluorescence Minus One (FMO) controls were strictly utilized for all multi-color panels to define precise positive gates, especially for markers with continuous expression distributions such as CD80, CD86, and MDSC-related markers.

For surface staining, approximately 1 × 10^6^ cells per sample were first incubated with an anti-mouse CD16/32 antibody to block Fc receptors, and then stained with a cocktail of fluorescently conjugated antibodies for 20 min at 4 °C in the dark. Fluorescence Minus One (FMO) controls and single-staining compensation beads were utilized to ensure precise gating and correct for spectral overlap between multiple fluorophores. The antibodies used were: CD3-APC/Cy7 (BioLegend, #100330), CD4-BV421 (BioLegend, #100438), CD8-PerCP/Cy5.5 (BioLegend, #100734), F4/80-APC (BioLegend, #123116), CD206-PE (BioLegend, #141706), CD86-PE/Cy7 (BioLegend, #105014), NKp46-FITC (BioLegend, #137605), CD11b-BV510 (BioLegend, #101263), Ly6G-Alexa Fluor 700 (BioLegend, #127622), Ly6C-BV605 (BioLegend, #128035). NK cells were identified as CD3- NKp46+. MDSCs were defined as CD11b+ Ly6G+ Ly6C+. The detailed gating strategy for each immune cell subset is provided in Supplemental Figure S8.

For intracellular Foxp3 staining (BioLegend, #320105), cells were fixed and permeabilized using the Foxp3/Transcription Factor Staining Buffer Set (eBioscience) according to the manufacturer’s instructions. Data were acquired on an LSRFortessa flow cytometer (BD Biosciences) and analyzed using FlowJo software (v10.8).

TUNEL staining.

Apoptotic cells within tumor tissues were detected using the One Step TUNEL Apoptosis Assay Kit (Beyotime, China). In brief, deparaffinized and rehydrated tumor sections were treated with Proteinase K for antigen retrieval. The sections were then incubated with the TUNEL reaction mixture for 60 min at 37 °C in a humidified dark chamber. Following this, the nuclei were counterstained with DAPI. Apoptotic cells (green fluorescence) were visualized and imaged under a fluorescence microscope (Leica DMi8).

### Quantification and statistical analysis

All quantitative results are expressed as the mean ± standard deviation. The appropriate statistical method was selected based on the experimental design. Specifically, comparisons between two independent groups were conducted using an unpaired, two-tailed Student’s t-test. When comparing the means of three or more groups, one-way analysis of variance (ANOVA) was employed, with Bonferroni’s correction applied for post-hoc multiple comparisons. All statistical calculations and graphing were executed using GraphPad Prism software (version 5.0, GraphPad Software, USA). A P-value of less than 0.05 was considered statistically significant, with specific levels denoted as *P < 0.05, **P < 0.01, and ***P < 0.001. Nonsignificant outcomes are indicated as NS.

## Results

### Low-dose SM enhances apoptotic activity in BC cells through indirect stimulation of the cGAS–STING pathway in endothelial cells

**Fig. 1:**
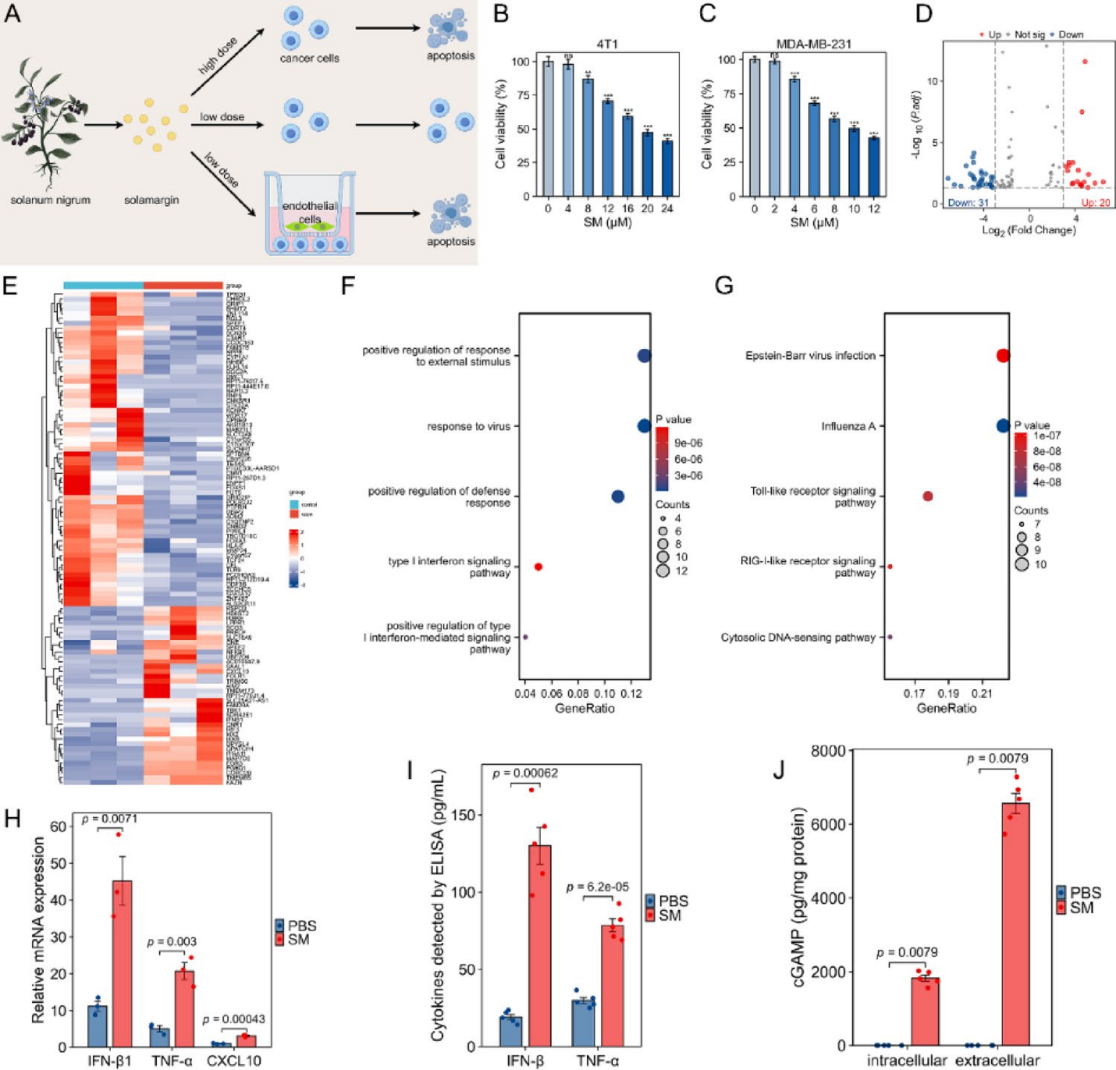
Low-dose solamargin (SM) induces breast cancer cell apoptosis indirectly by activating the cGAS–STING pathway in endothelial cells (HUVECs). **a** Schematic model illustrating the indirect mechanism. **b**,** c** Viability of (b) 4T1 and (c) MDA-MB-231 cells treated with a gradient concentration of SM (mean ± SD, *n* = 3). NS denotes not significant, **P < 0.01, ***P < 0.001 vs. control group (one-way ANOVA with Bonferroni’s test). **d** Volcano plot of differentially expressed genes in HUVECs. **e** Heatmap of the top 50 differentially expressed genes. **f**,** g** Enrichment analysis of DEGs based on (f) Gene Ontology (GO) and (g) KEGG pathways. **h** qRT-PCR analysis of STING-dependent cytokine genes (mean ± SD, *n* = 3). (unpaired two-tailed t-test). **i** ELISA measurement of IFN-β and TNF-α in co-culture medium (mean ± SD, *n* = 5). (unpaired two-tailed t-test). **j** ELISA quantification of cGAMP levels (mean ± SD, *n* = 5). (unpaired two-tailed t-test)

SM, a natural alkaloid from Solanum nigrum, has documented anticancer activity against hepatic carcinoma and NSCLC [13]; however, its effect on breast cancer (BC) remained unexplored. Initially, we assessed its direct cytotoxicity and found that the half-maximal inhibitory concentrations (IC₅₀) for 4T1 and MDA-MB-231 TNBC cells were 17.122 µg/mL and 8.866 µg/mL, respectively (Fig. 1a-c). Intriguingly, when we employed a non-cytotoxic low-dose SM in a co-culture system with HUVECs, it significantly induced apoptosis in the BC cells, whereas the same dose had no effect on BC cells in monoculture (Figure S1). This key observation suggested that the pro-apoptotic effect was indirectly mediated by endothelial cells.

To investigate the underlying mechanism, we performed transcriptomic analysis on SM-treated HUVECs. This revealed a set of differentially expressed genes (DEGs) (Fig. 1d, e). Subsequent KEGG and GO enrichment analyses indicated that these DEGs were significantly associated with the type I interferon (IFN) signaling pathway and the cytosolic DNA-sensing pathway (Fig. 1f, g). The cGAS–STING pathway is a well-established master regulator that bridges cytosolic nucleic acid sensing with the induction of type I IFN and related inflammatory responses. The concurrent enrichment of both these downstream functional categories in our transcriptomic data provided compelling preliminary evidence, leading us to hypothesize that low-dose SM triggers the activation of the cGAS–STING pathway in HUVECs.

We next sought experimental validation. qRT-PCR analysis confirmed a marked upregulation of downstream effector genes, including *IFN-β1*, TNF-α, and CXCL10 (Fig. 1H). Consistently, ELISA measurements demonstrated a substantial increase in the secretion of IFN-β and TNF-α proteins into the culture medium (Fig. 1i). Furthermore, as a direct readout of cGAS activity, we observed a significant elevation in both intracellular and extracellular cGAMP levels (Fig. 1j).

Taken together, these data demonstrate that low-dose SM activates the cGAS–STING pathway in endothelial cells, prompting the release of proinflammatory cytokines which, in turn, mediate the indirect apoptosis of breast cancer cells.

### Characterization and In Vivo Tumor Targeting of Au@PEG-SM

**Fig. 2:**
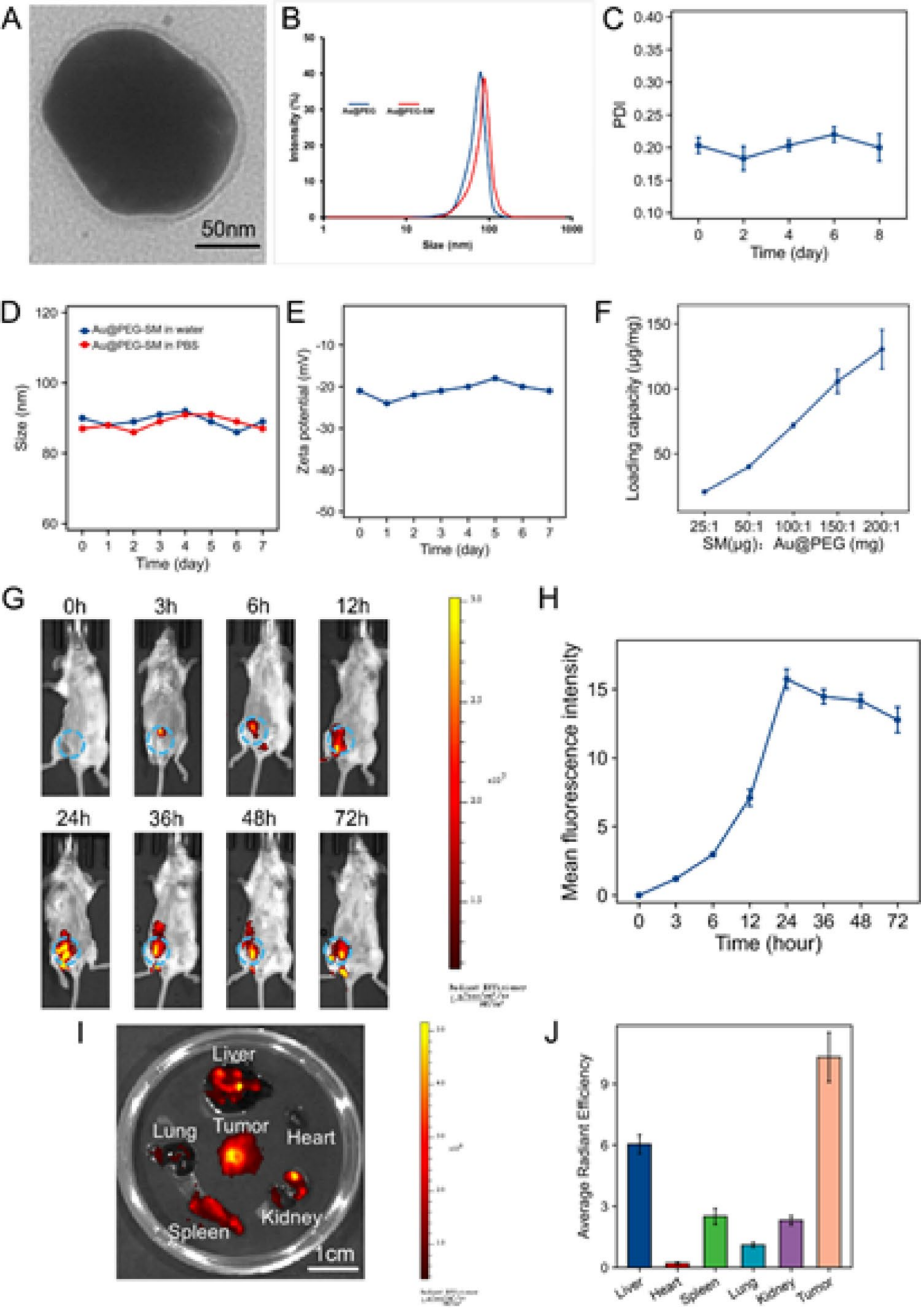
ynthesis, characterization, and in vivo targeting profile of Au@PEG-SM. **a** Representative TEM micrograph of Au@PEG-SM. Scale bar, 50 nm. **b** Hydrodynamic diameter distribution of Au@PEG and Au@PEG-SM. Data are representative of three technical replicates. **c** Polydispersity index (PDI) of Au@PEG-SM in PBS (*n* = 3 independent preparations). **d**,** e** Stability of Au@PEG-SM in water and PBS over 7 days, assessed by monitoring hydrodynamic diameter (d) and zeta potential (**e**). Data points show mean ± SD (*n* = 3 independent samples). **f** Drug loading efficiency at different SM: Au@PEG mass ratios. Data are mean ± SD (*n* = 3 independent experiments). **g** Longitudinal in vivo fluorescence imaging of 4T1 tumor-bearing mice after intravenous injection of Au@PEG-SM-DiD, **h** Quantification of time-dependent tumor accumulation from (**G**). Data are mean ± SD (*n* = 3 mice). **i** Ex vivo fluorescence images of dissected organs and tumors at 24 h post-injection. **j** Quantitative analysis of ex vivo fluorescence signals in harvested tissues. Data are mean ± SD (*n* = 3 mice). All quantitative data are presented as mean ± SD. The sample size (n) refers to biological replicates unless otherwise specified

Leveraging the excellent photothermal conversion efficiency and biocompatibility of AuNPs, we synthesized Au@PEG-SM, a nanoconstruct designed for tumor-targeted combination therapy. The AuNP core was modified with polyethylene glycol (PEG) to improve physiological stability, prolong systemic circulation, and enhance tumor accumulation through the EPR effect. Transmission electron microscopy (TEM) confirmed the spherical morphology of Au@PEG-SM (Fig. 2a), while dynamic light scattering (DLS) measurements showed a hydrodynamic diameter of 86.5 ± 6.2 nm with a narrow size distribution (Fig. 2, c). Furthermore, both the particle size and zeta potential (ranging from − 20 to − 25 mV) remained stable over 7 days in aqueous and physiological buffers, demonstrating the colloidal stability of the formulation (Fig. 2, e). We also optimized the drug loading capacity, achieving an encapsulation efficiency of 72.86% ± 3.62% at an SM-to-Au@PEG mass ratio of 100:1 (Fig. 2f).

To validate the photothermal functionality of Au@PEG-SM, we irradiated the nanoparticles at different concentrations (27 and 270 µg/mL) with an 808 nm laser (1 W cm⁻²). As expected, the solution temperature increased by 5.8 °C and 6.9 °C, respectively, whereas the control group showed a negligible temperature change (< 1 °C), confirming their strong photothermal conversion capability (Figure S2).

We next evaluated the in vivo targeting efficiency and biodistribution of Au@PEG-SM. Following intravenous administration in 4T1 tumor-bearing mice, fluorescence imaging revealed rapid and sustained accumulation of the nanoparticles at the tumor site, reaching a maximum at 24 h post-injection (Fig. 2g, h). Consistently, ex vivo imaging of resected tissues 24 h after injection showed significantly higher fluorescence intensity in tumors compared to major organs, underscoring the excellent tumor-targeting specificity of the nanoconjugate (Fig. 2i, j).

To provide precise quantitative evidence of the systemic circulation and targeting efficiency, we further analyzed the pharmacokinetics and biodistribution of Au@PEG-SM using ICP-MS. The blood concentration-time curve displayed a typical two-phase clearance pattern with a prolonged circulation half-life (t1/2 ≈ 8.5 h). Notably, a substantial concentration of gold (8.4 ± 1.2 µg/mL) was maintained in the bloodstream at 24 h post-injection, providing a sustained supply for continuous tumor enrichment (Figure S3). Quantitative biodistribution analysis at 24 h post-injection corroborated the fluorescence data, revealing high gold accumulation in the tumor tissue (12.5 ± 1.8 µg/g). While uptake was observed in the liver (18.2 ± 2.4 µg/g) and spleen (15.6 ± 1.9 µg/g) due to reticuloendothelial system clearance, the gold concentrations in the heart (0.8 ± 0.2 µg/g) and lungs (2.1 ± 0.5 µg/g) remained extremely low. Specifically, the accumulation in the kidneys was only 3.1 ± 0.6 µg/g, suggesting a low risk of renal impairment (Figure S4). These quantitative findings collectively confirm the superior tumor-targeting specificity and favorable pharmacokinetic profile of the Au@PEG-SM nanoplatform.

Finally, we assessed the biosafety profile of Au@PEG-SM. Histopathological examination (H&E staining) of major organs from treated mice showed no signs of inflammation or tissue injury. Moreover, blood routine and biochemical parameters remained within normal ranges, and no significant body weight loss was observed throughout the treatment period (Figure S5–S6), collectively indicating the favorable biocompatibility of Au@PEG-SM for in vivo application.

## Synergistic activation of the endothelial STING pathway and indirect tumoricidal effects

**Fig. 3:**
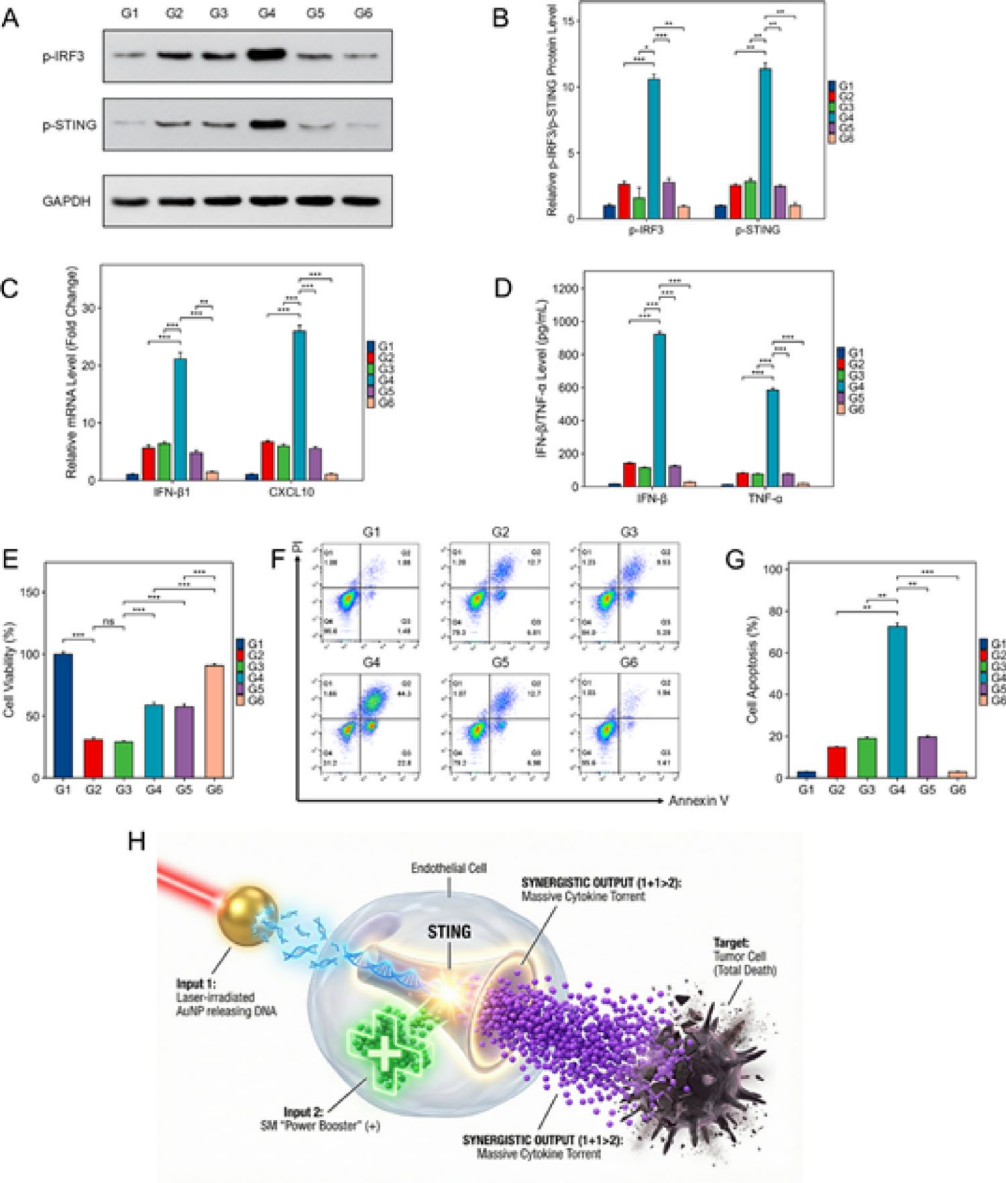
Validation of the synergistic endothelial STING activation and indirect tumoricidal mechanism. The experimental groups for pathway activation (**a**-**d**) are defined as: G1, Control; G2, PTT Ligand (DNA only); G3, SM only; G4, Synergy (SM + DNA); G5, Synergy + DNase I; and G6, Synergy + H-151. **a** Representative Western blot bands showing the expression of phosphorylated STING (p-STING, Ser366) and phosphorylated IRF3 (p-IRF3, Ser396) in vascular endothelial cells after 24 h of treatment, with GAPDH as the loading control. **b** Quantitative analysis of p-STING and p-IRF3 protein levels (normalized to GAPDH), demonstrating a significant 1 + 1 > 2 synergistic effect in the SM + DNA group. **c** RT-qPCR analysis of downstream effector genes, including IFN-β1 and CXCL10 mRNA levels. **d** ELISA quantification of secreted IFN-β and TNF-α in the cell culture supernatants. The experimental groups for cytokine neutralization assays (**e**-**f**) are defined as: G1, Control; G2, Model (CM-G4: conditioned media from G4-treated endothelial cells); G3, Isotype IgG (CM-G4 + Isotype IgG); G4, Anti-IFN-β (CM-G4 + neutralizing anti-IFN-β); G5, Anti-TNF-α (CM-G4 + neutralizing anti-TNF-α); and G6, Combined Neutralization (CM-G4 + Anti-IFN-β + Anti-TNF-α). **e** MTT assay evaluating the viability of 4T1 tumor cells after 24 h of incubation with the indicated CM and neutralizing antibodies. The synergistic killing effect was significantly rescued by cytokine neutralization.** f** Representative flow cytometry scatter plots of 4T1 cell apoptosis (Annexin V-FITC/PI staining) following various CM treatments.** g** Quantitative analysis of apoptotic 4T1 cells across the six groups as determined by flow cytometry.** h** Schematic illustration depicting the proposed mechanism: Au@PEG-SM-mediated PTT releases DNA ligands, which synergistically activate the sensitized STING pathway in endothelial cells, leading to a massive torrent of antitumor cytokines and subsequent tumor cell apoptosis. Data are presented as mean ± SD (*n* = 3). Statistical significance was calculated via one-way ANOVA: *P < 0.05, **P < 0.01, ***P < 0.001

To elucidate the molecular mechanism underlying the synergistic antitumor activity, we first investigated the activation of the STING pathway in vascular endothelial cells (G1–G6). Western blot analysis (Fig. 3a) and subsequent quantification (Fig. 3b, normalized to GAPDH) revealed that while DNA (G2) or SM (G3) alone induced moderate levels of protein phosphorylation, their combination (Synergy group, G4) resulted in a dramatic upregulation of phosphorylated STING (p-STING, Ser366) and IRF3 (p-IRF3, Ser396). Notably, the levels of p-STING and p-IRF3 in the synergy group were significantly higher than the sum of the individual treatments, demonstrating a potent 1 + 1 > 2 synergistic effect. Consistent with the activation of the STING-IRF3 axis, RT-qPCR analysis showed a massive increase in the mRNA expression of downstream effector genes, including IFN-β1 and CXCL10, in the synergy group (Fig. 3c).

Furthermore, ELISA results (Fig. 3d) confirmed the robust secretion of antitumor cytokines, including IFN-β and TNF-α, into the culture supernatant of the synergy-treated endothelial cells (G4). To verify the pathway specificity, we introduced DNase I (G5) to degrade the DNA ligands or H-151 (G6) to inhibit STING activity. Both interventions significantly abrogated the induction of cytokines, confirming that the synergistic response is both DNA-ligand dependent and STING-pathway dependent.

We next explored whether the endothelial secretome could indirectly eliminate tumor cells using conditioned media (CM) from G4 cells (CM-G4). 4T1 tumor cells were incubated with various CM and neutralizing antibodies (Fig. 3e-g, G1–G6). As shown by the MTT assay (Fig. 3e), the CM from the synergy group (Model group, G2) induced a significant decrease in 4T1 cell viability compared to the Control (G1). Notably, the addition of isotype IgG (G3) failed to rescue the cell viability, whereas the indirect tumoricidal effect was significantly rescued by neutralizing antibodies against IFN-beta (G4) or TNF-alpha (G5). The most pronounced rescue effect was observed in the combined neutralization group (G6), confirming that the indirect killing is specifically mediated by these cytokines. These findings were further corroborated by flow cytometric analysis (Fig. 3f, g), which showed that while the synergy-group CM triggered massive apoptosis in 4T1 cells, the blockade of these key cytokines significantly reduced the apoptotic rate. Collectively, these results demonstrate that the sensitized STING pathway in endothelial cells acts as a powerful molecular amplifier, converting synergistic signals into a massive torrent of cytokines that leads to tumor cell death (Fig. 3h).

### Au@PEG-SM Combined with PTT for Primary Tumor Treatment and cGAS–STING Pathway’s Activation

**Fig. 4:**
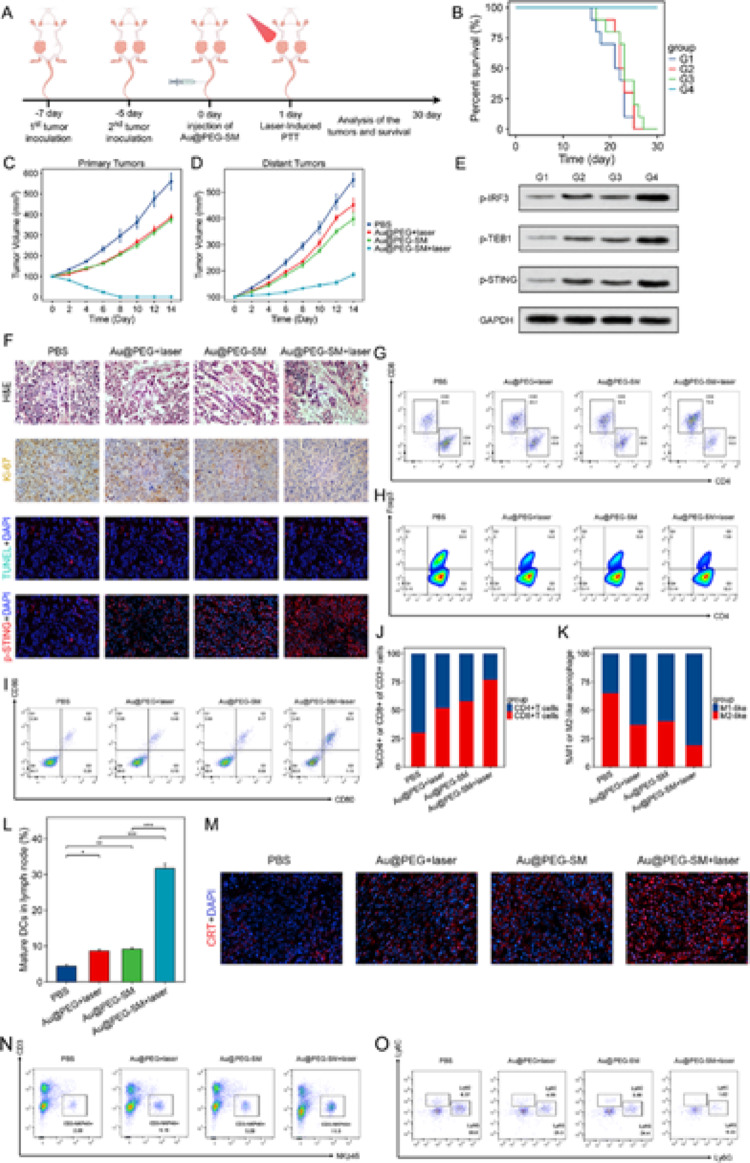
**a** Schematic of the therapeutic regimen in a bilateral 4T1 tumor model. **b** Survival curves of mice across different treatment groups (*n* = 10). G1: PBS (control), G2: Au@PEG + laser, G3: Au@PEG-SM, G4: Au@PEG-SM + laser. **c**,** d** Growth kinetics of laser-irradiated primary tumors (**c**) and non-irradiated distant tumors (**d**) (mean ± SEM, *n* = 6). **e** Western blot analysis of key proteins (p-STING, p-TBK1, p-IRF3) in the cGAS-STING pathway within primary tumors. **f** Histopathological and immunofluorescence analysis of primary tumors: H&E staining, Ki-67 (proliferation), TUNEL (apoptosis, green), and p-STING (activation, red) staining. Nuclei are counterstained with DAPI (blue). **g-i** Flow cytometric quantification of (**g**) CD8⁺ and CD4⁺ T cells, (**h**) regulatory T cells (Tregs), and (i) mature dendritic cells (CD86⁺CD80⁺) in tumor-draining inguinal lymph nodes. **(j**,** k)** Proportions of (**j**) tumor-infiltrating CD8⁺ T cells and (**k**) M1-like (F4/80⁺CD86⁺) versus M2-like (F4/80⁺CD206⁺) macrophages in primary tumors. **l** Quantitative analysis of mature dendritic cells in draining lymph nodes. **m** Immunofluorescence staining for calreticulin (CRT, red) in primary tumors, indicating immunogenic cell death. Nuclei are stained with DAPI (blue). **n** Flow cytometric quantification of tumor-infiltrating NK cells (CD3 minus NKp46 plus). **o** Flow cytometric quantification of myeloid-derived suppressor cells (MDSCs, CD11b plus Ly6G plus Ly6C plus) in primary tumors. *Data in c, d, l, n and o are presented as mean ± SD (*n* = 6). Statistical significance was determined by two-tailed Student’s t-test. *P < 0.05, **P < 0.01, ***P < 0.001

To evaluate the therapeutic efficacy of Au@PEG-SM combined with PTT, we established a bilateral 4T1 tumor model in BALB/c mice (Fig. 4a). Mice bearing tumors (~ 100 mm³) were randomly assigned to four groups (*n* = 10). Twenty-four hours after intravenous injection of Au@PEG-SM (10 mg SM/kg), primary tumors were irradiated with an 808 nm laser (1 W cm⁻², 15 min). Notably, the combination therapy group exhibited significantly improved survival compared to all control and monotherapy groups (Fig. 4b). Consistent with this, tumor growth curves showed that Au@PEG-SM plus PTT not only completely ablated the primary tumors but also markedly suppressed the growth of distant, non-irradiated tumors (Fig. 4c, d).

To elucidate the underlying immune mechanism, we first assessed activation of the cGAS–STING pathway. Western blot analysis of primary tumor tissues confirmed robust upregulation of phosphorylated STING, TBK1, and IRF3 in the combination therapy group (Fig. 4e). We further performed histopathological and immunofluorescence analyses on primary tumors collected two days post-treatment. H&E staining, Ki-67 immunohistochemistry (proliferation), and TUNEL assay (apoptosis) all indicated the most severe tumor damage in the combination group. Importantly, this enhanced cytotoxicity was accompanied by the highest levels of p-STING expression (Fig. 4f and S7), suggesting a link between STING activation and treatment efficacy.

Subsequent immune profiling of the primary tumor microenvironment revealed significant remodeling. Flow cytometry analysis showed that combination therapy led to a substantial increase in tumor-infiltrating CD8⁺ T cells and a concurrent decrease in regulatory T cells (Tregs) (Fig. 4g, h, j). In parallel, we observed a shift in macrophage polarization, characterized by an increased ratio of M1-like to M2-like macrophages (Fig. 4k and S8–9), indicating a reversal of immunosuppression. Furthermore, the combination treatment significantly promoted the recruitment of NK cells (CD3 minus NKp46 plus) into the tumors (Fig. 4n). Conversely, the population of myeloid-derived suppressor cells (MDSCs, CD11b plus Ly6G plus Ly6C plus), which are major contributors to the immunosuppressive microenvironment, was notably reduced in the synergy group (Fig. 4o). These results, together with the modulation of T cells and macrophages, demonstrate a comprehensive reprogramming of the tumor immune landscape.

To investigate how local treatment elicits systemic antitumor immunity, we analyzed dendritic cell (DC) maturation in tumor-draining lymph nodes. A significantly higher proportion of mature DCs was detected in the combination therapy group (Fig. 4i, l). We hypothesized that this effect stems from ICD induced in the primary tumor. Supporting this, ELISA measurements showed that serum levels of IFN-β and TNF-α were highest in the combination therapy group (Figure S10). Furthermore, immunofluorescence staining and semi-quantification of calreticulin (CRT), a hallmark of ICD, confirmed its peak exposure in the combination therapy group (Fig. 4m and S11). Collectively, these findings indicate that the combination of Au@PEG-SM and PPT induces potent ICD, which promotes DC maturation and triggers a systemic immune response capable of controlling distant tumor growth.

### Au@PEG-SM–Mediated PTT Induces Immune Responses in Distant Tumors

**Fig. 5:**
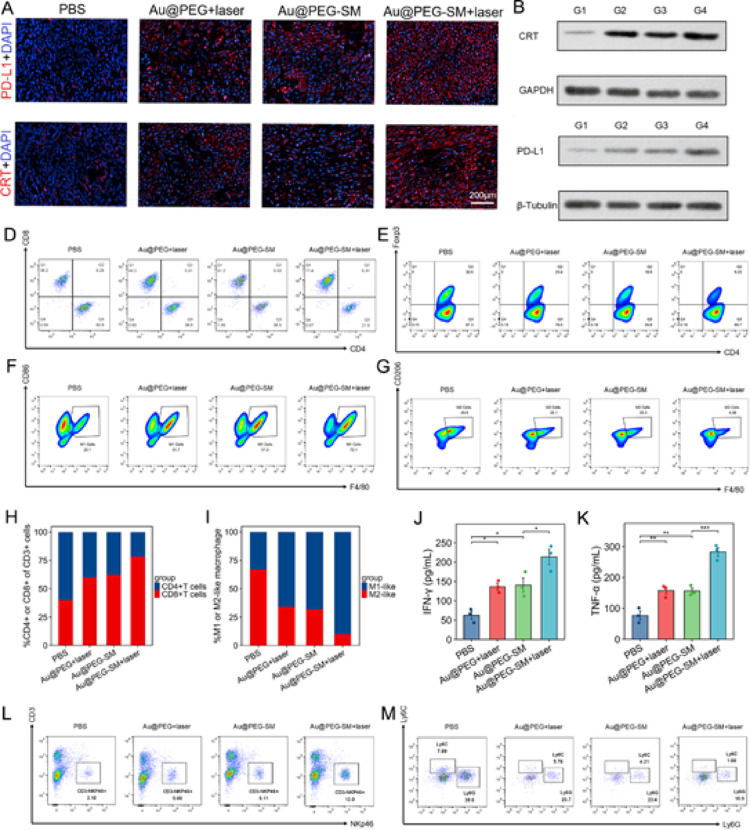
Au@PEG-SM-mediated PTT induces systemic immune responses in distant tumors.** a** Immunofluorescence staining of p-STING (upper) and calreticulin (CRT, lower) in distant tumors. Red, target protein; blue, DAPI (nuclei). **b**,** c** Western blot analysis of CRT (**b**) and PD-L1 (**c**) protein expression levels in distant tumors across treatment groups. G1: PBS; G2: Au@PEG + laser; G3: Au@PEG-SM; G4: Au@PEG-SM + laser. **d-g** Flow cytometric quantification of immune cell populations in distant tumors:** d** CD8⁺ and CD4⁺ T cells,** e** CD4⁺FoxP3⁺ regulatory T cells (Tregs),** f** M1-like macrophages (F4/80⁺CD86⁺), and** g** M2-like macrophages (F4/80⁺CD206⁺). **h**,** i** Ratios of (**h**) CD8⁺ to CD4⁺ T cells and** i** M1-like to M2-like macrophages in distant tumors. **j**,** k** Cytokine levels of (j) IFN-γ and (k) TNF-α in distant tumor homogenates, measured by ELISA. **l** Flow cytometric quantification of NK cells (CD3-NKp46+) and **m** myeloid-derived suppressor cells (MDSCs, CD11b+Ly6G+Ly6C+) in distant tumors. *Data in d-k, l and m are presented as mean ± SEM (*n* = 3 biologically independent samples). Statistical significance was determined by one-way ANOVA with Tukey’s post hoc test for multiple comparisons. *P < 0.05, **P < 0.01, **P < 0.001

To elucidate the systemic mechanism by which Au@PEG-SM–based combination therapy inhibits distant tumor growth, we analyzed non-irradiated distant tumors harvested two days post-treatment. Histological examination (H&E) and Ki-67 staining revealed the highest degree of necrosis and apoptosis, along with the most substantial suppression of proliferation, in the combination therapy group (Figure S12). Furthermore, mirroring the findings in primary tumors, distant tumors from this group exhibited the most robust activation of the cGAS–STING pathway and the highest levels of calreticulin (CRT) exposure, a key marker of immunogenic cell death (Fig. 5a, b and S13–14).

To gain further insight into the immune microenvironment, we performed qRT-PCR analysis of cGAS–STING–related genes. The expression of TNF-α, IFN-β1, CXCL9, CXCL10, ICAM-1, and VCAM-1 was most pronounced in the combination therapy group (Figure S15). Functionally, TNF-α and IFN-β1 are pivotal antitumor cytokines. Meanwhile, the interferon-inducible chemokines CXCL9 and CXCL10, by engaging the CXCR3 receptor, are crucial for recruiting activated CD8⁺ T cells and NK cells into tumors, thereby bridging innate and adaptive immunity [14]. Additionally, the adhesion molecules ICAM-1 and VCAM-1 facilitate the firm adhesion and transendothelial migration of immune cells within the tumor microenvironment [15–17].

We then focused on the interplay between the cGAS–STING pathway and immune checkpoint regulation, as PD-L1 expression is known to be modulated by this pathway. Prior studies have shown that PD-L1 deficiency can enhance cGAS–STING activity and IFN-β production [18], while radiotherapy-induced STING activation upregulates PD-L1 in hepatocellular carcinoma [19]. Similarly, in TNBC, cannabidiol promotes PD-L1 expression via the cGAS–STING pathway to enhance anti-PD-L1 efficacy [20]. These reports collectively suggest that cGAS–STING activation is a double-edged sword, potentiating antitumor immunity while simultaneously inducing PD-L1–mediated immune evasion.

Consistent with this paradigm, Western blot analysis confirmed that PD-L1 protein levels were most significantly upregulated in distant tumors from the combination therapy group (Fig. 5c). Concurrently, flow cytometric analysis revealed a substantial increase in CD8⁺ T cell infiltration and M1/M2 macrophage ratio, alongside a reduction in Tregs, within these distant tumors (Fig. 5d–i). Crucially, the combination treatment also elicited a significant increase in the infiltration of NK cells (CD3-NKp46+) in the abscopal tumors (Fig. 5l). In contrast, the level of immunosuppressive MDSCs (CD11b +Ly6G+Ly6C+) was markedly downregulated (Fig. 5m), indicating a systemic relief of immune suppression. Moreover, ELISA of tumor homogenates detected the highest concentrations of the effector cytokines IFN-γ and TNF-α in the combination group (Fig. 5j, k).

Based on these findings, we propose a mechanistic hypothesis: although the local combination therapy successfully initiates a potent systemic immune response—recruiting cytotoxic T cells and activating the tumor microenvironment—the concomitant STING-driven upregulation of PD-L1 on distant tumor cells likely impedes T cell–mediated cytotoxicity. This compensatory immune resistance mechanism may explain why the therapy significantly suppresses, but does not fully eradicate, distant tumors, highlighting a rationale for combining this strategy with PD-L1 checkpoint blockade.

### Au@PEG-SM–Mediated PTT Combined with Anti–PD-L1 Treatment for Distant Tumors

**Fig. 6:**
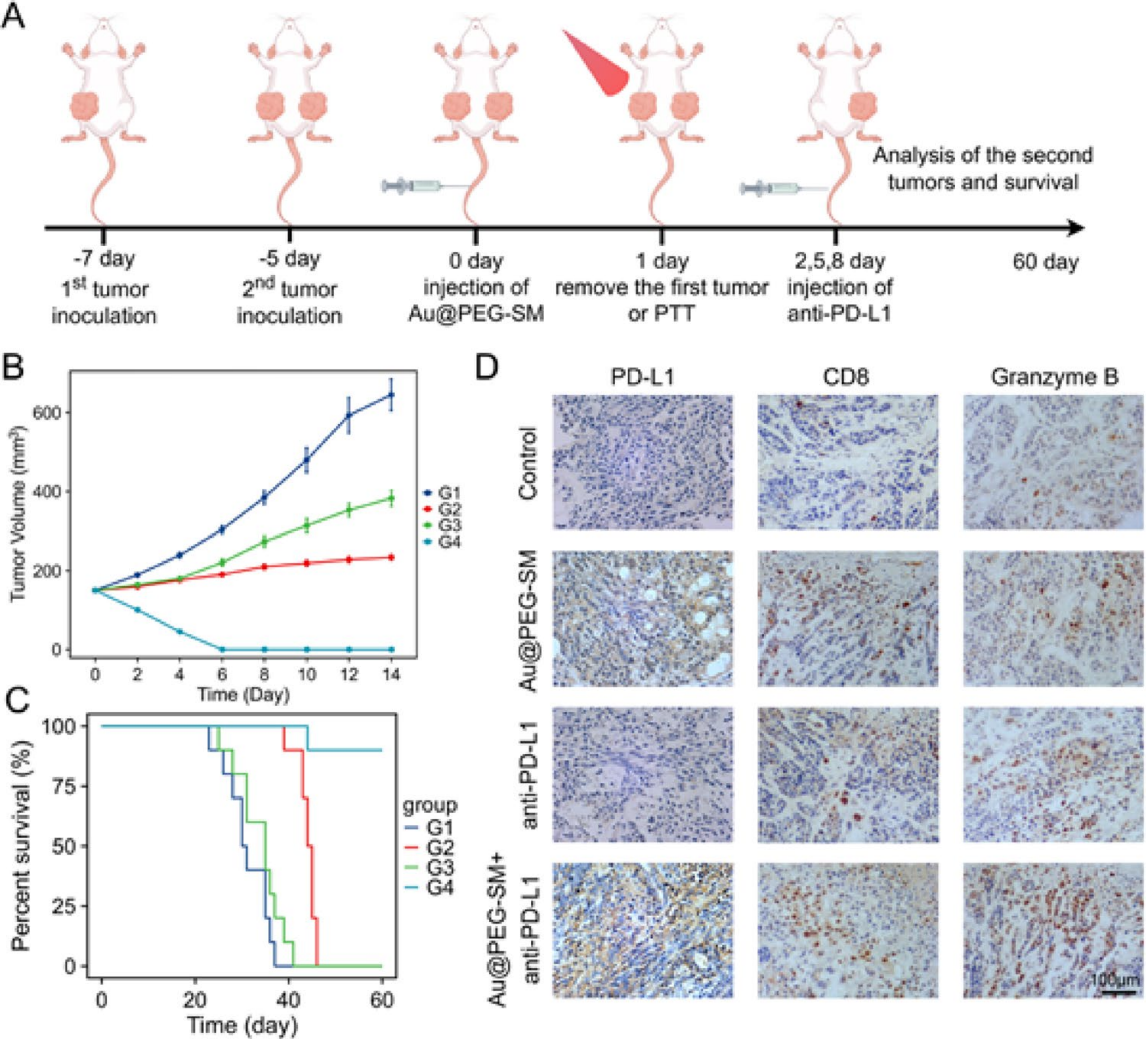
Combinatorial therapy of Au@PEG-SM-mediated photothermal therapy and anti-PD-L1 checkpoint blockade controls distant tumor growth. **a** Schematic diagram of the therapeutic schedule for distant tumor treatment. **b** Growth curves of distant tumors across different treatment groups (*n* = 6 mice per group). G1: Surgery; G2: Au@PEG-SM + laser; G3: Surgery + anti-PD-L1; G4: Au@PEG-SM + laser + anti-PD-L1. Data are presented as mean ± SEM. **c** Kaplan-Meier survival curves of mice bearing distant tumors (*n* = 10 per group). **d** Representative immunohistochemical staining of PD-L1, CD8 (T cells), and Granzyme B (GZMB, cytotoxicity marker) in distant tumor sections. Scale bar: 100 μm.

Next, we investigated whether the efficacy of Au@PEG-SM–based PTT could be enhanced by combination with anti–PD-L1 checkpoint blockade. A bilateral 4T1 tumor model was established, and mice were randomized into four treatment groups (*n* = 10). Twenty-four hours after intravenous injection of Au@PEG-SM (10 mg SM/kg), primary tumors were either surgically resected or subjected to PTT (808 nm laser, 1 W cm⁻², 15 min). Subsequently, mice received intraperitoneal injections of anti–PD-L1 antibody (200 µg) or an isotype control on days 2, 5, and 8 post-initial treatment (Fig. 6a).

The therapeutic outcomes revealed a clear synergistic effect. While Au@PEG-SM–mediated PTT or anti–PD-L1 monotherapy showed limited efficacy in controlling distant tumor growth, their combination led to the complete eradication of distant tumors in all treated mice (Fig. 6b). Consistently, this combination group also exhibited the most significant survival benefit (Fig. 6c).

To delineate the underlying immune mechanisms, we performed immunohistochemical analysis of distant tumors harvested on day 9. As anticipated, Au@PEG-SM–mediated PTT robustly upregulated PD-L1 expression in distant tumors, while the addition of anti–PD-L1 antibody effectively counteracted this induction (Fig. 6d and S16). Furthermore, the PTT group showed enhanced CD8⁺ T cell infiltration in distant tumors compared to the surgery-only group. Most importantly, the combination of PTT and anti–PD-L1 therapy resulted in the highest levels of Granzyme B expression, indicating that PD-L1 blockade reinvigorated cytotoxic T lymphocytes (CTLs), enabling them to effectively kill tumor cells and achieve complete distant tumor clearance (Fig. 6d and S16)

.

### Au@PEG-SM–Mediated Therapy in Conjunction with Anti–PD-L1 Inhibitors for Recurrence

**Fig. 7:**
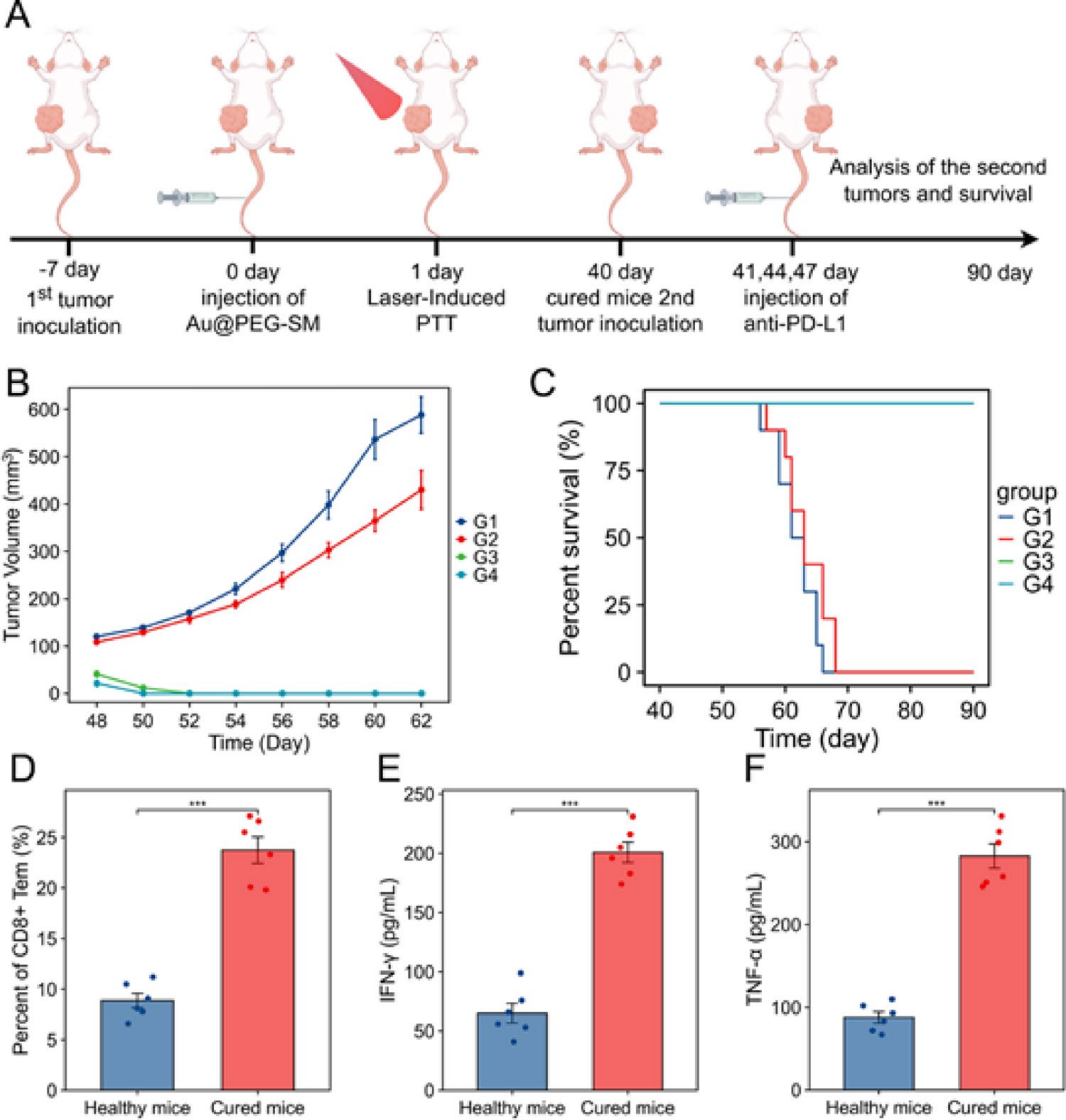
Au@PEG-SM-mediated photothermal therapy combined with anti-PD-L1 prevents breast cancer recurrence and establishes long-term immunity. **a** Experimental timeline for tumor rechallenge and immune memory assessment.**b** Tumor growth curves following tumor rechallenge in different treatment groups (*n* = 6 mice per group). G1: Naive mice; G2: Naive mice + anti-PD-L1; G3: Cured mice; G4: Cured mice + anti-PD-L1.**c** Kaplan-Meier survival curves after tumor rechallenge (*n* = 10 mice per group).**d** Flow cytometric analysis of splenic T cell populations in naive and cured mice on day 40. **e**,**f** Serum levels of IFN-γ (**e**) and TNF-α (**f**) in naive and cured mice on day 40. *Data in** B**-**F** are presented as mean ± SEM (*n* = 6 for B, D-F; *n* = 10 for C). Statistical significance was determined by two-tailed Student’s t-test (D-F). ***P < 0.001.

We next evaluated the capacity of Au@PEG-SM–mediated PTT to generate durable antitumor memory immune responses. In a rechallenge model established 40 days after primary tumor eradication, cured and naive mice received 4T1 cells and either anti–PD-L1 or control antibody (Fig. 7a). Remarkably, cured mice exhibited robust resistance to tumor rechallenge independent of PD-L1 blockade, as evidenced by suppressed tumor growth and enhanced survival (Fig. 7b, c), indicating the establishment of functional immune memory. Immunological analysis revealed that this protection coincided with a significant expansion of splenic CD8⁺ effector memory T cells (CD3⁺CD8⁺CD62L⁻CD44⁺) and elevated systemic levels of TNF-α and IFN-γ in cured mice (Fig. 7d-f). These results demonstrate that Au@PEG-SM–mediated PTT induces a persistent antitumor immune state capable of mediating long-term protective immunity against tumor recurrence.

.

## Discussions

The therapeutic landscape for TNBC is often constrained by the toxicities associated with potent chemotherapeutics and the dose-limiting side effects of natural monomers [21]. Our study addresses this challenge by utilizing a gold nanoparticle-based platform to deliver low-dose SM, shifting the therapeutic focus from direct tumor cytotoxicity to a sophisticated modulation of the tumor microenvironment. This strategy is consistent with the evolving paradigm of “dose-reduction and effect-enhancement” in nanomedicine, where targeted delivery systems leverage the tumor’s physiological characteristics to achieve superior efficacy with minimal systemic exposure.

A central finding of this work is the pivotal role of tumor-associated endothelial cells in mediating SM-induced antitumor effects. While the importance of the cGAS-STING pathway in the tumor microenvironment has been established in existing oncology research [22, 23], our data highlights that low-dose SM can act as a potent STING agonist specifically within endothelial cells. This observation aligns with recent paradigms in vascular oncology, which suggest that the tumor endothelium functions not merely as a physical barrier but as a sophisticated immunological rheostat capable of initiating T-cell infiltration [16].This triggers a secretory switch in the endothelium, characterized by the robust release of IFN-β and TNF-α. Unlike strategies that aim to directly ablate the tumor vasculature, our approach leverages the biosynthetic capacity of endothelial cells to transform the primary tumor site into an immunogenic hub. This finding extends the understanding of endothelial-immune crosstalk, suggesting that the vascular lining can be reprogrammed to actively participate in tumor cell apoptosis rather than merely serving as a passive conduit for nutrients.

The synergy between SM-mediated biochemical activation and gold nanoparticle-mediated PTT is crucial for overcoming the immunosuppressive nature of TNBC. While PTT is known to induce immunogenic cell death through thermal stress [24], the co-delivery of SM provides a secondary, reinforcing signal through the cGAS-STING axis. The enhancement of calreticulin exposure and antigen release observed in our study suggests that SM acts as a potent “immunological adjuvant” for thermal ablation, a concept supported by recent studies on multimodal nanoplatforms that maximize the “in situ vaccine” effect of PTT [25]. This intense local immunogenic event effectively bridges the gap between local ablation and systemic surveillance, as evidenced by the enhanced maturation of dendritic cells in draining lymph nodes.

However, the systemic activation of the cGAS-STING pathway presents a biological paradox. Our findings indicate that while this pathway recruits cytotoxic T lymphocytes to distant sites, it simultaneously drives a compensatory upregulation of PD-L1 on tumor cells. This “immune cloaking” is a well-documented resistance mechanism where Type I interferons—while initially immunostimulatory—eventually feedback to stabilize PD-L1 expression through the JAK-STAT axis, thereby dampening the sustained effector function of CD8 + T cells [14]. By integrating anti-PD-L1 blockade, we successfully uncoupled the immunostimulatory benefits of STING activation from its immunosuppressive side effects. This tri-modal strategy successfully eradicated both established distant lesions and reinvigorated the effector function of CTLs.

Regarding long-term protection, the Au@PEG-SM platform demonstrated the capacity to establish a reservoir of CD8-positive effector memory T cells. In our murine rechallenge model, this memory response provided sustained protection against tumor recurrence. It is important to note, however, that while these results are promising in a controlled experimental setting, the translation of such immune memory to the clinical heterogeneity of human TNBC requires further investigation. Factors such as the timing of PTT and the durability of STING-mediated memory in humans remain subjects for future study.

In summary, Au@PEG-SM offers a unique paradigm for TNBC therapy by combining low-dose traditional Chinese medicine monomer delivery with physical ablation. By targeting the endothelial cGAS-STING pathway and combining it with checkpoint blockade, this nanoplatform provides a robust strategy to overcome both local tumor growth and systemic recurrence.

## Conclusions

In summary, this study presents an innovative Au@PEG-SM nanoplatform that achieves targeted codelivery of low-dose SM and gold nanoparticle (AuNP)-mediated PTT for TNBC treatment. The system not only leverages the EPR effect for tumor-specific accumulation but also unveils a novel cascade mechanism validated by rigorous loss-of-function studies. Our results demonstrate that low-dose SM sensitizes the cGAS-STING pathway in endothelial cells, which, upon synergistic activation by PTT-released DNA, triggers a potent secretome-mediated indirect tumoricidal effect.

Comprehensive immune profiling reveals that this combination strategy profoundly remodels the tumor microenvironment by recruiting innate NK cells and adaptive CD8 plus T cells, while significantly reducing the infiltration of immunosuppressive MDSCs and regulatory T cells in both primary and distant tumors. Furthermore, the therapy induces robust ICD, promotes dendritic cell maturation, and establishes durable antitumor immune memory. By mechanistic inhibition and cytokine neutralization assays, we confirmed the specific causal link between STING-driven secretome and tumor apoptosis. Our work provides a safe, efficient, and systemically active multimodal therapeutic paradigm with strong translational potential for TNBC treatment.

## Data Availability

The datasets used and/or analyzed during the current study are available from the corresponding author on reasonable request.
